# Temporal variation and potential origins of atmospheric speciated mercury at a remote island in South China Sea based on two-year field measurement data

**DOI:** 10.1038/s41598-021-84434-z

**Published:** 2021-03-11

**Authors:** Ming-Jie Yeh, Chung-Shin Yuan, Kuo-Ning Hung, Iau-Ren Ie, Cheng-En Lee, Kuan-Chen Chiang, Ker-Yea Soong

**Affiliations:** 1 Institute of Environmental Engineering, National Sun Yat-Sen University, Kaohsiung City, Taiwan, ROC; 2 Institute of Marine Biology, National Sun-Yat Sen University, Kaohsiung City, Taiwan, ROC

**Keywords:** Climate sciences, Environmental sciences

## Abstract

This study explored the temporal variation, gas-particle partition, and potential origins of atmospheric speciated mercury at a remote island in the South China Sea. Two-year data of three mercury species was measured at the Taiping Island. Air masses were clustered into five transport routes (A-E) to resolve the potential origins of atmospheric mercury. Field measurement showed that the concentration of gaseous elemental mercury (GEM) (1.33 ± 0.52 ng/m^3^) was close to the GEM background level of Northern Hemisphere, while those of GOM and PHg were 13.39 ± 3.58 and 94.33 ± 30.25 pg/m^3^, respectively. Both regular and intensive samplings concluded a consistent trend of higher mercury level in winter and spring than that in summer and fall. GEM dominated atmospheric mercury in all seasons (86.2–98.5%), while the highest partition of particle-bound mercury (PHg) was observed in winter (13.8%). The highest GEM concentrations were observed for Route A originating from central China and western Taiwan Island, and followed by Routes D and E from the Philippines, Malaysia, and Indonesia, while the lowest concentrations of GEM were observed for Routes B and C originating from North China, Korea, and Japan. Most importantly, high correlation of GEM versus levoglucosan and K^+^ in PM_2.5_ (r = 0.764 and 0.758, *p* < 0.01) confirmed that GEM was mainly emitted from biomass burning sources at the surrounding countries.

## Introduction

In the natural environments, mercury maintains dynamic balance in various media including air, soil, and water^1^. Ocean plays an important role as mercury source or sink in the mercury cycle^2^. Atmospheric mercury not only influences local ambient air quality but could also move to the leeward lands and waters via long-range transport, causing a global environmental impact on bioaccumulation of mercury in marine organisms^2–4^.

In addition to natural sources such as volcano irruption and forest fires, anthropogenic sources and industrial activities are major sources of atmospheric mercury. Mercury-containing substances that originally existed in the earth’s minerals were massively released to the atmosphere mainly through high-temperature combustion, non-ferrous metal smelting, and cement production processes^5^. Mercury and its derivatives have been treated as a worldwide hazardous pollutant due to its persistent, bioaccumulation, and toxic properties, which have been proven as a threat to human health^6^. Moreover, it can be spread worldwide through long-range transport in the atmosphere and causes global environmental issue^7^, which has been claimed as the second world environmental issue following global warming and has drawn much attention from governments and non-governmental organization (NGO) in the world^6^.

In the atmosphere, mercury exists in three main forms, namely gaseous elemental mercury (GEM), gaseous oxidized mercury (GOM), and particle-bound mercury (PHg)^8–10^. The former two forms are collectively referred as total gaseous mercury (TGM), and all three are referred as total atmospheric mercury (TAM)^11^. Among them, GEM is the dominant species in the atmosphere both locally and globally^12^. Due to its low reactivity and poor water solubility, GEM is not easily removed by wet and dry depositions, and thus most likely to perform global atmospheric circulation in the atmosphere for over 0.5–2 years^1,13–15^. GOM is a highly chemically reactive species that can be dissolved in water or attached on the surface of suspended micro-tissues, and has a residence time of only a few hours to weeks in the atmosphere^1,3,16^. Unlike GEM, GOM can be easily scavenged by wet and dry depositions due to its high water solubility, thus its transport distance in the atmosphere is only about several hundred kilometers^14,17^. PHg is characterized as particle bound mercury and has the atmospheric residence time of only a few days, which can be commonly removed by both dry and wet depositions and eventually falls to the lands and the waters^18–20^.

The spatial distribution of GEM is in the global scale, while GOM is more likely locally distributed. In terms of regional and global impacts, GEM tends to transport long distance in the atmosphere, while GOM commonly falls in a relatively short distance by dry and wet scavenging^2,21^. Settling down to the lands or the waters, GOM might cause even more serious local environmental problems than GEM. In terms of deposition characteristics, GEM can be settled down via dry deposition or chemically reacts with non-carbon substances to form inorganic mercury and its deposition flux is very slow. Oppositely, GOM can be settled down to the earth surface much rapidly via both wet and dry scavenging^3^.

**Figure 1:**
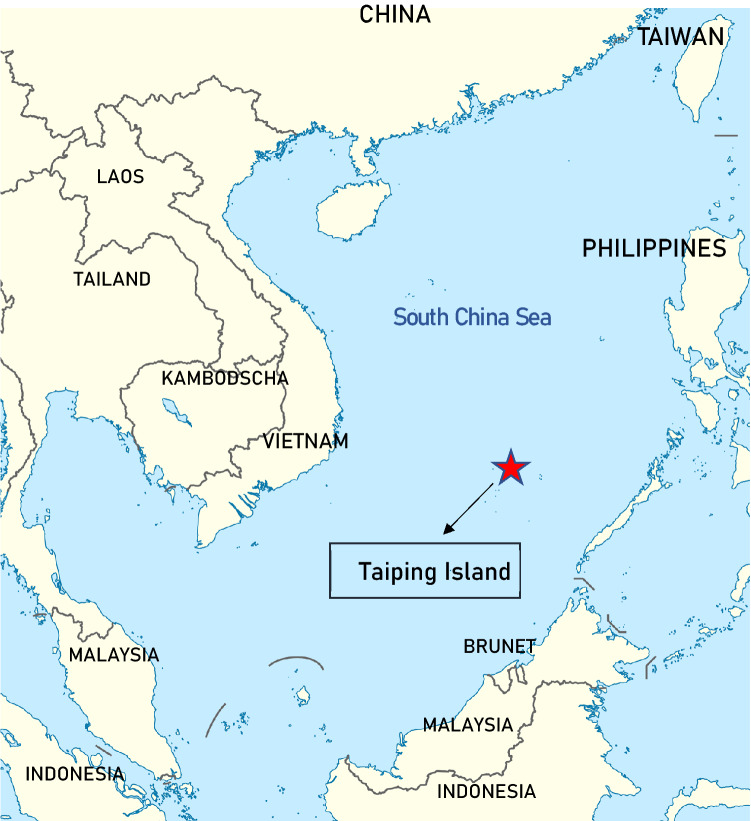
Location of atmospheric speciated mercury sampling site at the Taiping Island in the South China Sea.

Under the influences of prevailing Asian monsoons, mercury originated from the highly urbanized and industrialized areas in the Northeast Asia and from the biomass burning frequently occurring in the Southeast Asia can be commonly transported by a continental outflow toward the leeward marine regions^22^. One of the major leeward marine regions is the SCS where is the largest marginal sea in Asia. Among hundreds of islands, Taiping Island is located in the center of the SCS (see Fig. 1) and is suitable as a remote marine background site for the measurement of atmospheric speciated mercury in the East Asia.

Previous studies reported that atmospheric speciated mercury along with other air pollutants (i.e. PM_2.5_) could be transported easterly from the East Asian continent to the leeward areas of Korea Peninsula, Japan Islands, Hawaii Islands, and even the West Coast of the United States^23–25^. Additionally, atmospheric speciated mercury could be transported southerly from the Northeastern Asia to the Taiwan Strait and even the northern SCS^22,26^. The emissions of mercury-containing pollutants from East Asian as a continental outflow to the leeward marine regions have been increasing in the past decades. Although previous relevant studies have focused on the effects of oversea and sea-land transport for atmospheric speciated mercury^27–29^, the investigation on the temporal variation and potential sources of atmospheric speciated mercury in the northern SCS is still limited^22,27,30^. In particular, the quantitative description on the cross-boundary transport of atmospheric speciated mercury toward the Taiping Island located at the central part of SCS has not yet been thoroughly investigated in the past.

Accordingly, this study attempts to conduct a mercury sampling campaign at the Taiping Island for two consecutive years in order to investigate the temporal variation and gas-particle partition of atmospheric speciated mercury at the Taiping Island in the central SCS. Further investigation on the resolution of potential origins of atmospheric mercury in the upwind regions was also undertaken by backward trajectory simulation and statistical correlation with the fingerprints of biomass burning.

## Methodologies

### Sampling protocol

In this study, field sampling of atmospheric speciated mercury was conducted at a remote island, the Taiping Island, in the central SCS from September 2017 to August 2019. The Taiping Island is the largest natural island with fresh water in the Nansha Islands, which is approximately 1600 km from the southern tip of the Taiwan Island and about 300 km away from the Palawan Island, the Philippines. The area of the Taiping Island is approximately 0.51 km^2^ and has no permanent residents except for the national coast guard of Taiwan. As illustrated in Fig. 1, the Taiping Island is located at the center of the SCS with the longitude and the Latitude of 10° 22′ 38″ N and 114° 2′ 59″ E, respectively.

Three species of atmospheric mercury (TGM, GOM, and PHg) were simultaneously sampled at a platform of a tower about 7 m above the ground for two consecutive years. The sampling of speciated mercury in the atmosphere was divided into two phases in the present study. The first phase was conducted to collect 12-h mercury samples for consecutive seven days (i.e. intensive sampling). Daytime sampling was conducted from 8:00 am to 8:00 pm, while nighttime sampling was conducted from 8:00 pm to 8:00 am on the next day. The second phase was undertaken to collect 24-h mercury sample in an interval of twelve days (i.e. regular sampling). Both intensive and regular samplings of atmospheric speciated mercury were employed in order to investigate the temporal variation of atmospheric speciated mercury at the Taiping Island in the SCS. However, during the entire sampling periods, the field sampling of atmospheric speciated mercury at the Taiping Island was not conducted in the fall of 2017 for regular sampling as well as in the summer of 2018 for both intensive and regular samplings solely due to military restriction reasons.

In addition to the field measurement of atmospheric speciated mercury at the Taiping Island, the present study simultaneously sampled marine fine particles (PM_2.5_) at the same sampling site^31^. The sampling of PM_2.5_ was conducted by using quartz fibrous filters with a PQ-200 sampler for further measuring the mass concentration and chemical composition of PM_2.5_. PM_2.5_ filters were initially subjected to ultrasonic vibration for at least 4 h and filtration, and then analyzed with a high performance ion chromatography (HPIC) for the specific measurement of levoglucosan and potassium ion (K^+^).

### Sampling methods of TGM, GOM, and PHg

A standard method for “sampling and analysis of total gaseous mercury (TGM) and particle-bound mercury (PHg) in ambient air” promulgated by National Institute of Environmental Analysis (NIEA A304.10C), mainly adopted from U.S. Environmental Protection Agency (EPA Method IO-5), was applied for the field measurement of atmospheric speciated mercury in this study. The sampling of atmospheric mercury in the gaseous and particulate phases involves the use of gold-coated bead traps and quartz fibrous filters, respectively. The amalgamation of gaseous mercury requires a low air flowrate of 0.3 L/min to allow the effective adsorption of TGM onto the gold surface. However, low levels of PHg require a much higher air flowrate (10 L/min) to ensure a sufficient amount of particles to be collected by quartz fibrous filter for the following PHg measurement. Moreover, an annular denuder with inner wall coating with potassium chloride (KCl) was applied to adsorb GOM via molecular diffusion mechanism^32,33^. Similar to PHg, it also required a much higher air flowrate (10 L/min) to ensure a sufficient amount of GOM to be collected by the annular denuder for the following GOM measurement.

**Figure 2:**
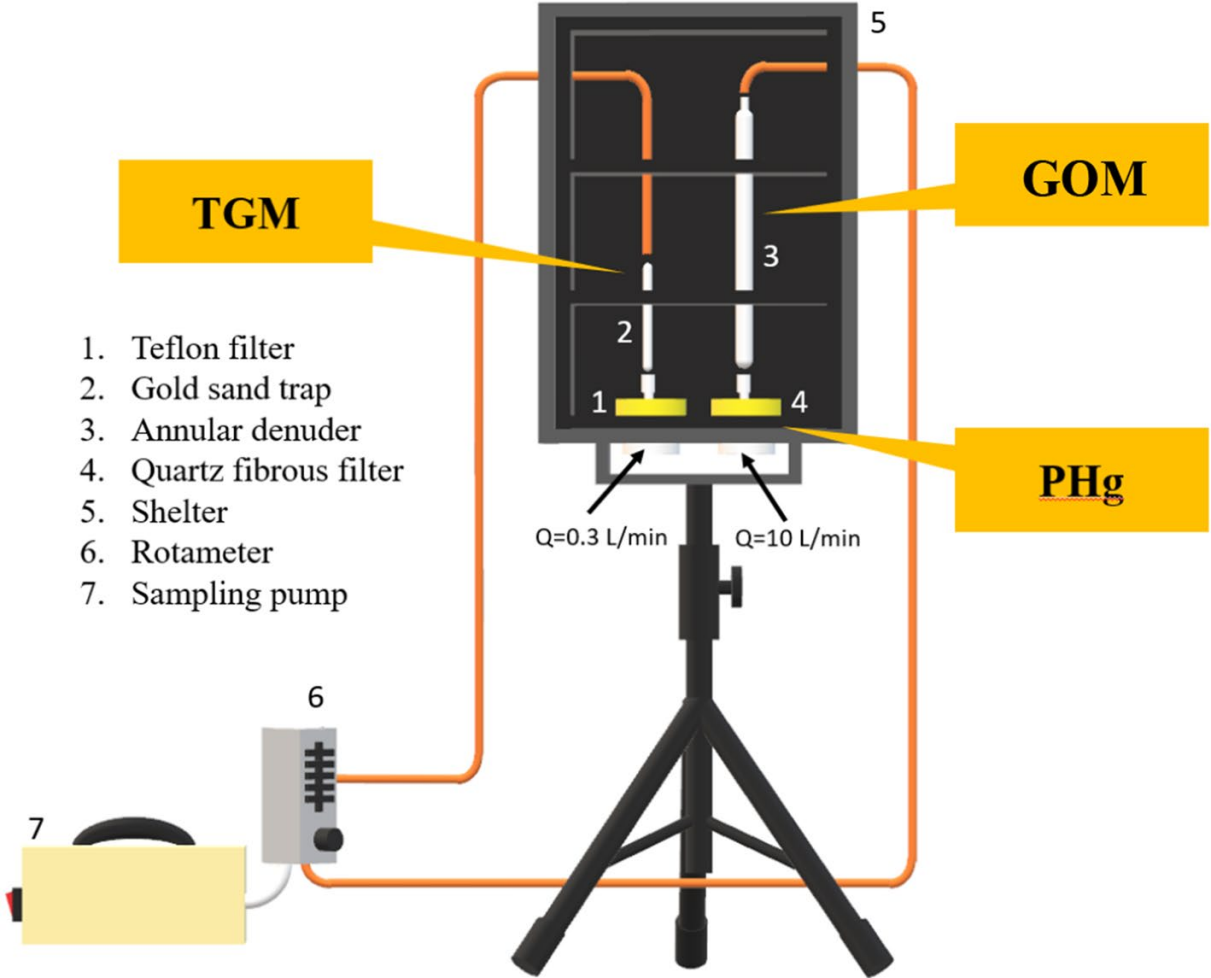
Schematic diagram of atmospheric speciated mercury sampling system for field measurement at the Taiping Island in the South China Sea.

Therefore, a separate atmospheric speciated mercury sampling system was self-designed for this particular study, as illustrated in Fig. 2, applying to collect atmospheric mercury in both gaseous and particulate phases. Most importantly, accurate air flowrate is crucial for sampling atmospheric speciated mercury, thus we conducted the calibration procedure for correcting the air flowrate of the sampling system periodically in order to accurately determine the concentrations of atmospheric speciated mercury.

### Analytical methods of TGM, GOM, and PHg

After sampling, the concentration of mercury in ambient air was further analyzed with a cold-vapor atomic fluorescence spectrometry (CVAFS). In addition to the direct sampling of TGM with a gold-coated sampling trap, GOM and PHg were initially expelled from annular denuder and quartz fibrous filer, respectively, and then adsorbed by the gold-coated sampling traps. The gold-coated sampling traps were heated at 450 °C for 2 h to release the adsorbed speciated mercury from the gold-coated beads. The desorbed mercury was then carried by an inert gas (Ar) to the CVAFS, known as the analytical trap. Mercury collected on the analytical trap was further thermally desorbed and entered into the CVAFS by Ar for further mercury analysis.

The analysis of mercury with the CVAFS was undertaken by the following three steps: (1) The Hg sampling (TGM) or adsorption (GOM/PHg) tube was thermally desorbed to release the adsorbed Hg from the gold surface with inert carrier gas (Ar); (2) The desorbed Hg molecules then entered into the CVAFS for detection; (3) The Hg molecules absorbed the incident ultraviolet (UV) light and emitted fluorescence that was converted to a voltage signal proportional to the amount of Hg by a photomultiplier detector and integrated the voltage peak area by an integrator.

Prior to conducting the chemical analysis of mercury species, calibration curves were initially prepared for the subsequent analysis of mercury concentration. In this study, two calibration curves for high- (ng) and low-level (pg) concentrations of mercury were prepared for GEM and GOM/PHg measurements, respectively (see Fig. S1). The determination coefficient (r^2^) of the calibration curves for the analysis of atmospheric mercury must be 0.99 or higher (r^2^ ≥ 0.99). Each of the points on the high- and low-level calibration curves predicted by its slope should be within 5 and 10% differentia of their true values, respectively. In this study, the r^2^ of the calibration curves for the analysis of GEM and GOM/PHg in the levels of ng and pg were 0.999 and 0.997, respectively, which met the quality assurance and quality control (QA/QC) requirement of r^2^ ≥ 0.99.

### Clustered transport routes and origin resolution

This study further applied the Hybrid Single Particle Lagrangian Integrated Trajectory (HYSPLIT) model developed by Air Resources Laboratory of National Oceanic Atmospheric Administration (NOAA), USA. It is a complete system for computing single air trajectories as well as complex transport and dispersion. One of the most common applications is the backward trajectory analysis which has been widely applied to identify the potential origins of polluted air masses^34,35^. When a high concentration level of a target air pollutant is measured in a specific time slot at the sampling site, it shows that a polluted air mass passes through the sampling site during that time slot. For this particular study, a Hybrid Single Particle Lagrangian Integrated Trajectory (HYSPLIT) model was applied to simulate the backward trajectories of air parcels starting from the Taiping Island at three selected heights (i.e. 100, 300, and 500 m) above the ground for 72-h transport of the air parcels, which were then plotted for the sampling days during the regular and intensive sampling periods. Backward trajectories and regional fire maps were further combined to identify the potential origins of atmospheric mercury with the 3-D wind field data.

Additionally, the measurement data collected by the NASA MODIS satellite was used to plot the fire map in the target region of East and South Asia. The fire spots on the earth surface were detected by using a multi-spectral sensor mounted on the MODIS satellite to analyze the middle-infrared and thermal infrared spectrum irradiated from the high-temperature burning sources, which can then be applied to locate the higher temperature areas over the ground surface and further determine the reasonable coordinates^36^.

## Results and discussion

### Temporal variation of GEM, GOM, and PHg concentrations

This study conducted both regular and intensive samplings of GEM, GOM, and PHg at the Taiping Island in the SCS and further measured their concentrations. Table 1 summarizes the seasonal average and standard deviation of GEM, GOM, and PHg concentrations at the Taiping Island for two consecutive years. The seasonal variation of three species of atmospheric mercury at the Taiping Island showed slight different. According to the average concentrations of atmospheric speciated mercury for both regular and intensive samplings in each season for two consecutive years, the seasonal average concentrations of GEM were ordered as: spring > winter > summer > fall, and those of GOM were ordered as: spring > summer > winter > fall, while those of PHg were ordered as: winter > spring > summer > fall (see Table 1). In summary, the average concentrations of GEM and PHg in the cold seasons (i.e. winter and spring) were always higher than those in the hot seasons (i.e. summer and fall). High concentrations of GEM and PHg observed in winter and spring were mainly attributed to the transport of upwind polluted air masses containing mercury towards the Nansha Island in the SCS via long-range transport. Unlike GEM and PHg, a different trend of seasonal average GOM concentration was observed at the Taiping Island. High concentrations of GOM in spring and summer (see Table 1) was mainly attributed to the oxidation of GEM to GOM in the atmosphere due to relatively high solar radiation and ambient air temperature in the marine boundary layer^9,37^.

**Table 1:** Comparison between intensive and regular samplings of atmospheric speciated mercury at the Taiping Island in South China Sea.

Years	Seasons	Sampling modes	n	GEM (ng/m^3^)	GOM (pg/m^3^)	PHg (pg/m^3^)
2017	Fall	Intensive	21	1.32 ± 0.11	10.87 ± 1.47	20.57 ± 10.23
		Regular*	0	–	–	–
2018	Winter	Intensive	21	1.31 ± 0.24	12.58 ± 2.93	149.52 ± 50.11
		Regular	24	1.56 ± 0.13	14.12 ± 1.51	71.44 ± 20.08
	Spring	Intensive	21	1.59 ± 0.10	17.01 ± 1.68	130.22 ± 19.98
		Regular	9	1.46 ± 0.09	13.84 ± 1.16	59.79 ± 10.33
	Summer	Intensive*	0	–	–	–
		Regular*	0	–	–	–
	Fall	Intensive	7	1.32 ± 0.10	10.05 ± 1.26	19.63 ± 9.51
		Regular	7	1.29 ± 0.08	9.67 ± 0.77	30.28 ± 10.05
2019	Winter	Intensive	7	1.23 ± 0.20	12.95 ± 2.39	128.94 ± 27.54
		Regular	8	1.28 ± 0.07	12.45 ± 1.14	140.48 ± 45.61
	Spring	Intensive	7	1.59 ± 0.10	16.67 ± 1.59	131.21 ± 10.86
		Regular	6	1.31 ± 0.19	15.42 ± 0.45	110.87 ± 10.24
	Summer	Intensive	7	1.31 ± 0.20	13.05 ± 1.55	50.39 ± 9.68
		Regular	7	1.43 ± 0.09	14.06 ± 2.02	68.49 ± 28.57
All Seasons		Intensive	91	1.33 ± 0.52	13.39 ± 3.58	94.33 ± 30.25
		Regular	61	1.43 ± 0.43	13.47 ± 5.74	68.18 ± 19.88

*GEM* gaseous elemental mercury, *GOM* gaseous oxidized mercury, *PHg* particle-bound mercury, *Spring* March–May, *Summer* June–August, *Fall* September–November, *Winter* December–February, *n* number of samples.

*The sampling of atmospheric speciated mercury was not undertaken in the fall of 2017 and the summer of 2018 due to military restriction.

Other possible reason for such seasonal trend was the burning of agricultural debris (i.e. biomass burning) in the areas surrounding the Taiping Island in the seasons of winter and spring. A huge amount of GEM and PHg could be emitted by biomass burning from the neighboring continents, causing a high concentration of GEM and PHg at the downwind Taiping Island. However, mercury originated from the northern upwind polluted regions might be too far to transport to the central SCS during the northeastern monsoon periods. During the long-range transport processes, they could be dispersed in the atmosphere and tended to deposit to the earth surface, both the lands and the seas, on their way to the downwind regions, resulting in relatively low concentration of mercury at the Taiping Island. As a result, the GEM and PHg sources in the neighboring countries could be much crucial for the contribution of GEM and PHg at the Taiping Island.

One interesting finding of this study was that the ambient air temperature and solar radiation in summer at the Taiping Island were always the highest; however, the concentration of GOM commonly oxidized from GEM was hardly noticeable in summer. It was attributed to the situation that the emissions of GEM from the neighboring areas might be insufficient amount which thus limited the effective chemical conversion of GEM to GOM in the atmosphere. As a result, the concentrations of GOM at the Taiping Island maintained at a relatively low level in summer compared to those in spring. Another possibility was that GOM formed in summer could be further reduced more back to GEM.

**Figure 3:**
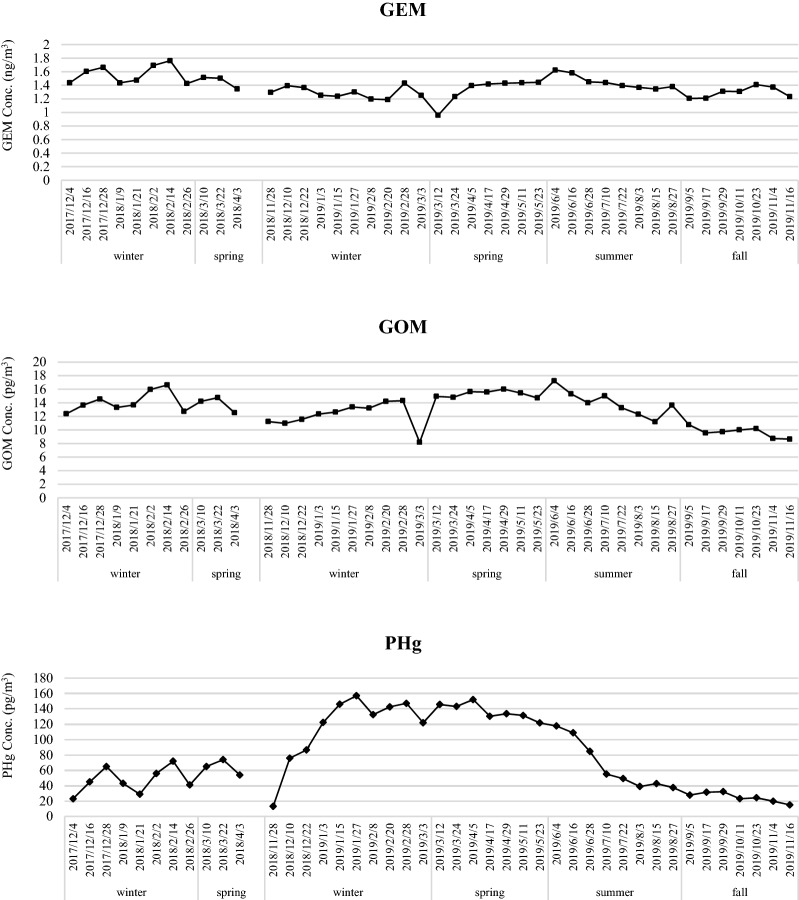
Temporal variation of atmospheric speciated mercury concentration for regular sampling conducted at the Taiping Island in the South China Sea.

**Figure 4:**
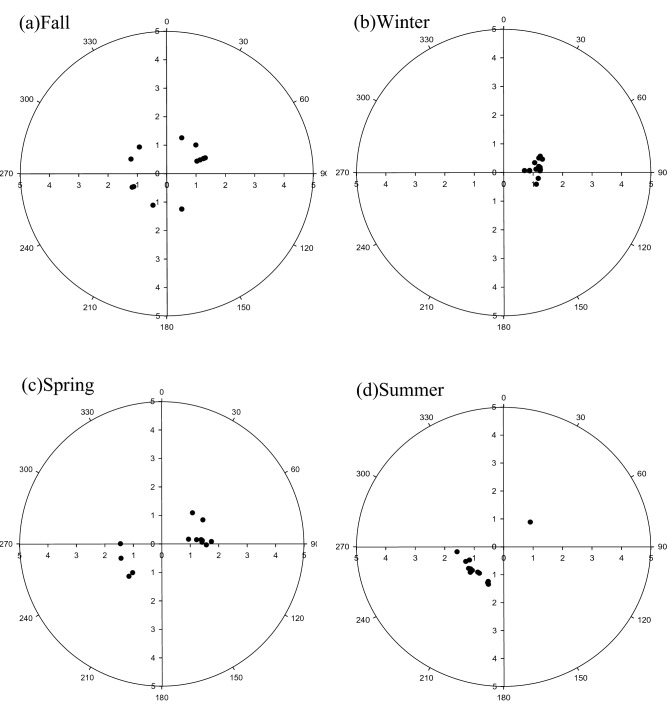
Pollution rose of GEM concentration at the Nansha Island.

This study further plotted the time series of atmospheric speciated mercury concentrations measured at the Taiping Island for regular sampling as depicted in Fig. 3. It illustrates that the GEM and GOM concentrations for the two-year regular sampling period did not show significant variation. Both regular and intensive sampling results demonstrated that the highest GOM and PHg concentrations were observed in winter and spring, while their lowest concentrations were found in summer and fall. However, the seasonal variation of PHg concentration appeared to be more obviously than that of GEM and GOM since PHg tended to fall down significantly during the long-range transport process, which might cause PHg unable to arrive at the Taiping Island. Additionally, high rainfall in summer and fall could scavenge particle-bound mercury down to the lands and waters during the long-range transport processes, resulting in low concentration of PHg observed at the Taiping Island in the SCS (Fig. 4).

### Gas-particle partition of atmospheric mercury

**Figure 5:**
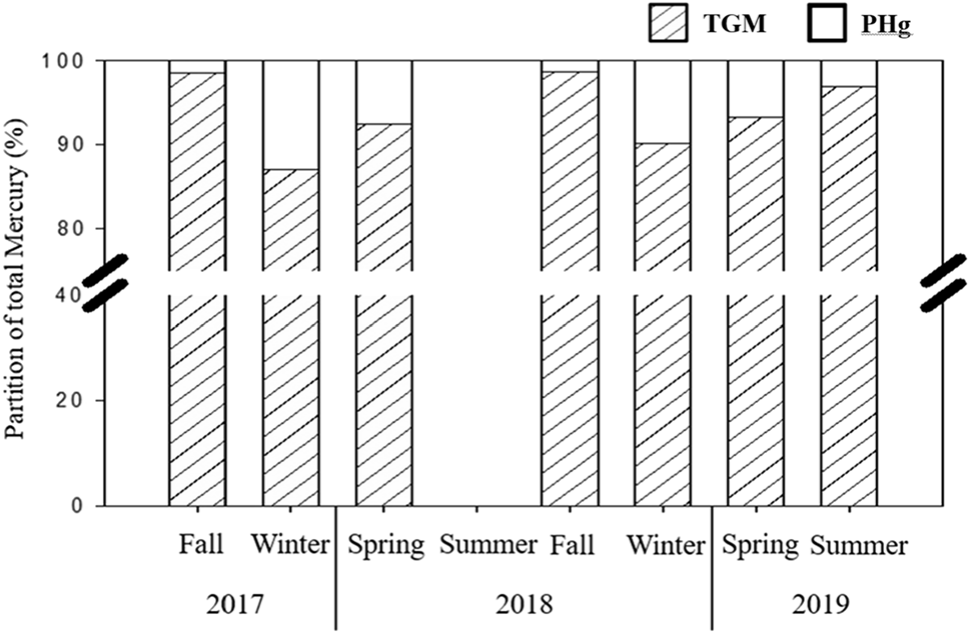
Gas-particle partition of atmospheric mercury at the Taiping Island for two-year measurement data.

The gas-particle partition of atmospheric mercury sampled at the Taiping Island was further investigated in this study. Fig. 5 illustrates the partition of gaseous and particulate phases of atmospheric mercury for two-year sampling periods. Similar to previous published literature^22,38–40^, the dominant phase of atmospheric mercury at the Taiping Island was gaseous mercury (TGM = GEM + GOM) (Table 1, Fig. 5). Moreover, a seasonal variation of gas-particle partition of atmospheric mercury was observed at the Taiping Island. The proportion of TGM in TAM (TGM/TAM) was 98.76% (fall), 90.17% (winter), 93.25% (spring), and 96.91% (summer), respectively, showing that the two-year average and standard deviation of TGM/TAM was 94.77 ± 3.83%. Additionally, the proportion of PHg in TAM (PHg/TAM) was 1.24% (fall), 9.83% (winter), 6.75% (spring), and 3.09% (summer) respectively, showing that the two-year average and standard deviation of PHg/TAM was 5.23 ± 3.83% (Table 1). It revealed that, in four seasons, the highest proportion of PHg in TAM was observed in winter and followed by spring, summer, and fall (Fig. 5). The highest portion of PHg/TAM in winter and spring implied that the mercury sources of Taiping Island came mainly from the upwind neighboring regions. The lowest portion of PHg/TAM in summer and fall was mainly due to the scavenging of particle-bound mercury by rainout and washout.

**Figure 6:**
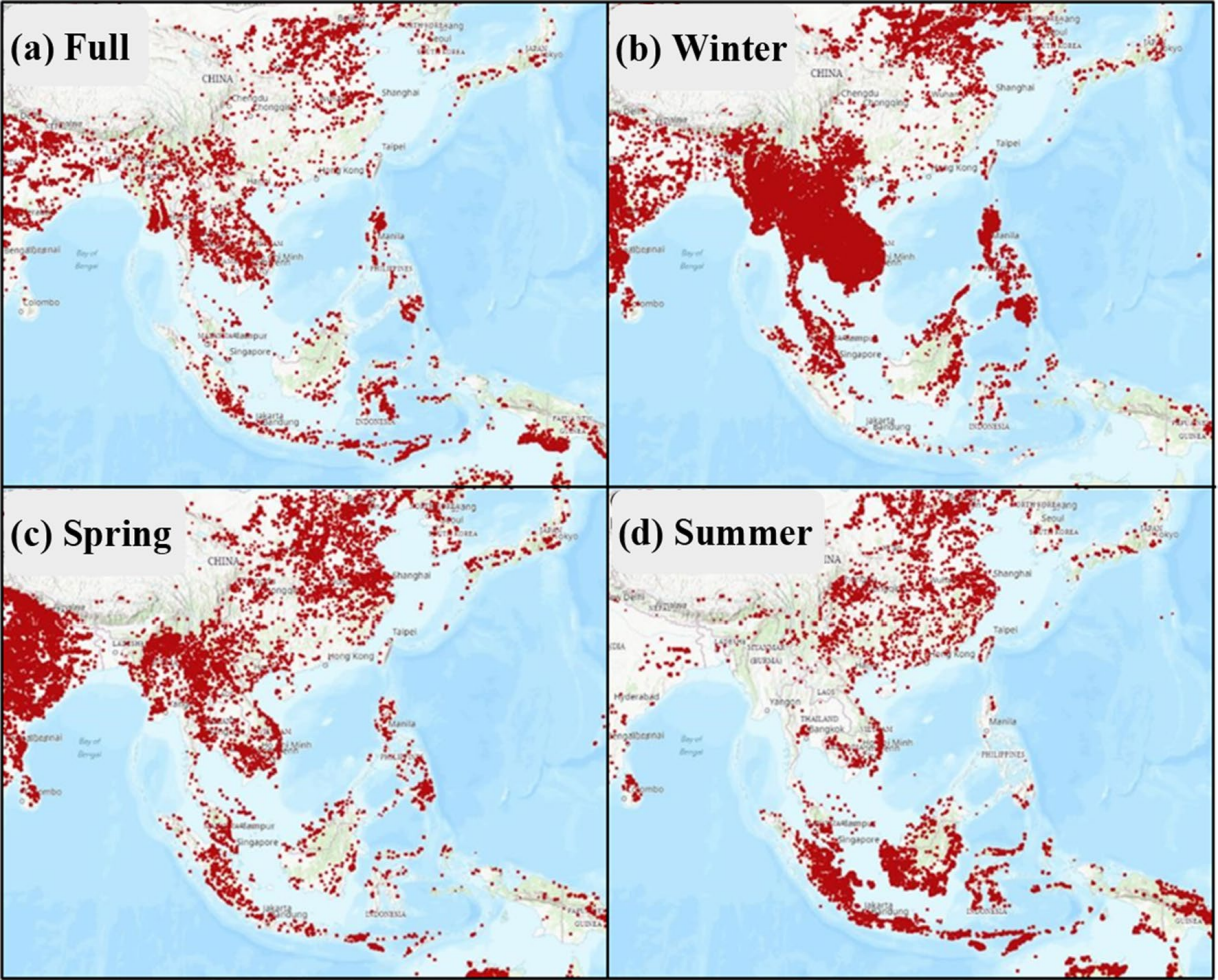
Fire maps in the East and South Asia measured by the NASA MODIS satellite.

According to the regional fires occurred in the East and South Asia in winter and spring (see Fig. 6b,c), a plenty of fire spots on the earth surface were observed in the Southeast Asia and mainland China, where a huge amount of mercury in three forms were emitted to the atmosphere, which can be further transported to the Taiping Island in the SCS. Although PHg tended to deposit gradually due to gravity forces during the long-range transport processes, the distance between the PHg emission sources and the Taiping Island might not be too far for particle-bound mercury transporting toward the Taiping Island, resulting in high PHg concentrations and mass ratios of PHg/TAM in winter.

As a matter of fact, there are no large-scale mercury sources in the vicinity of the Taiping Island. Only very few anthropogenic activities (e.g. vehicles, cargo ships, and fishing boats) were observed during the sampling periods. In addition, high TAM concentrations at the Taiping Island might be mainly attributed from the burning of plant and grass debris regionally. Moreover, the Taiping Island is located at the leeward region of the Philippines Islands while the prevailing northeastern monsoons were blown to the SCS in winter and spring. Another possibility was that both gaseous and particulate phases of mercury-containing pollutants emitted from slash-and-burn farming that frequently occurred in Southeast Asia could be highly probably transported to the Taiping Island in spring and summer.

### Potential origins of atmospheric mercury resolved from clustered transport routes

This section attempts to plot a series of regional fire maps in the East and South Asia to compare their spatial distribution in four seasons during the two-year sampling periods, which can be further applied to identify the potential origins of atmospheric mercury sampled at the Taiping Island for the following relevant discussion. The global fire maps have been widely used by numerous studies to locate the burning sources over the ground surface in a large geological scale^26^.

According to the fire maps in fall season, the fire spots (i.e. the sources of open burning) in the East and South Asia was evenly distributed in India, Indonesia, the Indochina Peninsula, North China, and the Philippines Islands. Although the fire spots in fall were not as dense as those in winter and spring, they were relatively stable and evenly distributed in the regions. In winter and spring, the distribution of fire spots highly concentrated in the Indochina Peninsula, India, Malaysia, the Philippines Islands, South China, Central China, and North China. As far as the winter season was concerned, there were many densely distributed fire spots covering the entire Indochina Peninsula, where a large number of biomass burning sources were commonly observed in winter. Moreover, the fire spots in the Philippines Islands were more densely distributed in winter than those in summer. Particularly, there was a violent trend for the distribution of open fires in the entire India in spring. The density of fire spots in the Indochina Peninsula, South China, and North China in spring were less than those in winter, and only sporadic open fires occurred in Central China. Spring is the most vigorous season for agricultural activities since many food and economic crops were cultivated in this season. Thus, spring was also the season for the burning of agricultural debris which frequently occurred in the Southeast Asia. In addition to the influences of biomass burning from mainland China and the Indochina Peninsula, the burning of agricultural debris was commonly observed in almost the entire Southeast Asia, particularly in Indonesia, resulting in the highest concentrations of atmospheric speciated mercury and levoglucosan in spring (see Tables 1 and S1). In summer, the open fires were evenly distributed in the mainland China, which was lower than those in winter and spring. On the contrary, more dense distribution of fire spots were observed in Indonesia and East Malaysia in the Southeast Asia (see Fig. 6), resulting in higher atmospheric mercury at the Taiping Island during the southwestern monsoon periods..

**Figure 7:**
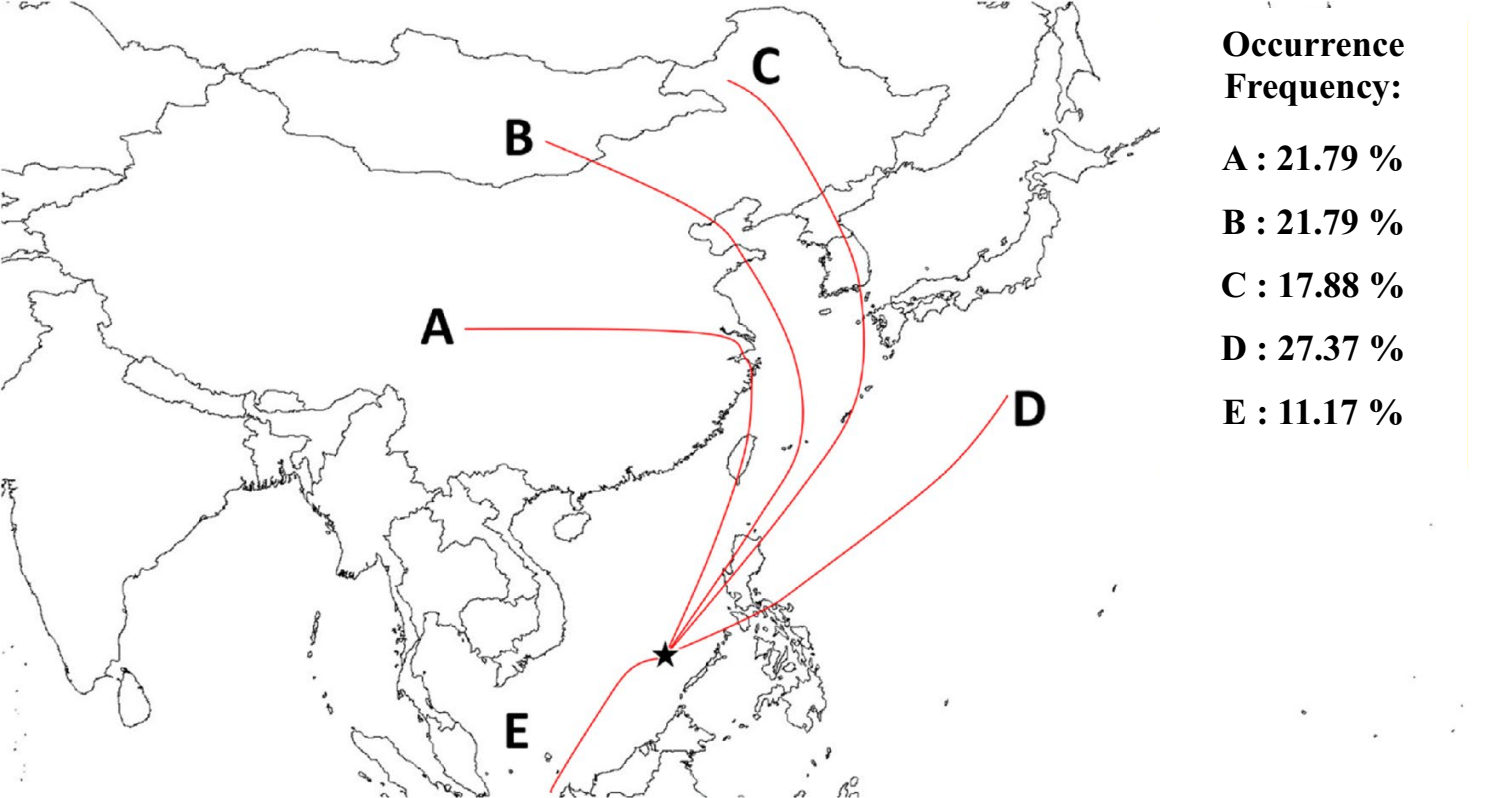
Clustered transport routes of air masses moving toward the Taiping Island in the South China Sea (★: Taiping Island).

Furthermore, this study applied a HYSPLIT model to simulate and plot the backward trajectories originating from the Taiping Island during the sampling periods. The backward trajectories of air masses were then clustered into five representative transport routes (Routes A-E) moving towards the Taiping Island as illustrated in Fig. 7. Route A was originated from Central China passing through the Yangtze River Delta (YRD) and moving along the West Taiwan Island. Route B originating from North China and Mongolia Plateau passed through metro Beijing and Tianjin of North China, and transported across the East China Sea (ECS) along the eastern waters of the Taiwan Island passing through North Luzon Island toward the Taiping Island. Route C was originated from Northeast China passing through Korea Peninsula and South Japan Islands, and transported across the ECS and North Luzon Island toward the Taiping Island. Route D transported southwesterly from the West Pacific Ocean through the metro Malena in the Central Philippines Islands toward the Taiping Island. Route E originating from East Malaysia and Indonesia in the Southeast Asia was transported northeasterly through the southern waters of the SCS toward the Taiping Island. The occurrence frequencies of Routes A through E were 21.59, 21.59, 20.45, 17.05, and 19.32%, respectively (see Table 2).

**Table 2:** Occurrence frequency and route-based average GEM concentration for different clustered transport routes moving toward the Taiping Island.

Transport routes*		A	B	C	D	E
Source regions		Central China	North China	Korea/Japan	West Pacific Ocean	South China Sea
Fall	Frequency (%)	23.81	23.81	19.05	19.05	14.28
	GEM (ng/m^3^)	1.31 ± 0.06	1.21 ± 0.08	1.18 ± 0.07	1.38 ± 0.03	1.41 ± 0.05
Winter	Frequency (%)	23.33	26.67	26.67	13.33	10.00
	GEM (ng/m^3^)	1.58 ± 0.13	1.30 ± 0.09	1.25 ± 0.09	1.28 ± 0.31	1.38 ± 0.27
Spring	Frequency (%)	30.43	26.09	26.09	13.04	4.35
	GEM (ng/m^3^)	1.66 ± 0.08	1.34 ± 0.09	1.33 ± 0.05	1.35 ± 0.10	1.36 ± 0.00
Summer	Frequency (%)	–	–	–	28.57	71.43
	GEM (ng/m^3^)	–	–	–	1.37 ± 0.06	1.47 ± 0.12
All seasons	Frequency (%)	21.59	21.59	20.45	17.05	19.32
	GEM (ng/m^3^)	1.54 ± 0.17	1.29 ± 0.12	1.26 ± 0.13	1.36 ± 0.18	1.45 ± 0.19

*The transport routes are clustered in Fig. 6.

As far as the three typical species of atmospheric mercury are concerned, the chemical activity and water solubility of GEM are much lower than other two species, which makes GEM to be much more difficultly scavenged from the atmosphere than GOM and PHg by dry and wet depositions. Consequently, GEM becomes the predominant species of atmospheric mercury in most of the atmospheric environments and can be distributed globally via long-range transport. The concentrations of GEM at the Taiping Island for different transport routes are shown in Table 2.

The highest GEM concentrations were observed in Route A and followed by Routes D and E, while the lowest GEM concentrations occurred in Routes B and C. Among the five distinct transport routes, Route A originating from Central China showed a curved pathway passing through the YRD and West Taiwan Island (see Fig. 7), both are highly industrialized and urbanized regions, with the highest occurrence frequency in the seasons of spring and fall (Table 2). This specific transport route brought a huge amount of mercury toward the SCS, and thus resulted in the highest yearly averaged GEM concentrations of 1.54 ± 0.17 ng/m^3^ with the seasonal range of 1.31–1.66 ng/m^3^ at the Taiping Island (Table 2). Other two northern transport routes (Routes B and C) originating from either Northeast China or Mongolia plateau passed through North China, Korean Peninsula, South Japan Islands, the East China Sea, and North Luzon Island, with high occurrence frequencies in winter and spring. These two transport routes showed the lowest seasonal averaged concentrations of GEM ranged from 1.18 to 1.34 ng/m^3^ (Table 2). It was most likely attributed to the dilution effect of mercury in the atmosphere owing to the effective dispersion for the longest distance transported from its origins to the Taiping Island. Route D originating from the West Pacific Ocean passed through the Central Philippines Islands, particularly the highly urbanized metro Manila, the capital city of the Philippines, and then transported toward the Taiping Island (see Fig. 7). This transport route (Route D) ranked the third highest yearly averaged GEM concentrations ranging from 1.28 to 1.38 ng/m^3^ (Table 2) were mainly attributed from both industrial emissions and agricultural activities including fuel and biomass burning in the Philippines Islands, which resulted in the increase of GEM concentration at the Taiping Island. Another possible reason was the much shorter distance from its origins in the Philippines Islands to the Taiping Island, which dramatically dissipated the dilution effect over the dispersion of GEM during the transport process. Ranking as the second highest yearly averaged GEM concentration of 1.45 ± 0.19 ranging from 1.36 to 1.47 ng/m^3^ at the Taiping Island (Table 2), Route E was speculated to be caused by the biomass burning in the Sumatra and Kalimantan Islands, with the highest occurrence frequency in summer (see Table 2). Regional fire maps confirmed that the fire spots were concentrated on the Sumatra and Kalimantan Islands in the south during the summer sampling period (see Fig. 6). Similar to Route D, Route E also had relatively short distance from its sources in the Sumatra and Kalimantan Islands to the Taiping Island while compared to other two northern transport routes (Routes B and C).

This study further summarized the occurrence frequencies of transport routes and their route-based average GEM concentrations at the Taiping Island by combining the backward trajectories and the corresponding GEM concentration in all seasons (see Table 2). It showed that the two year-round frequencies of different transport routes were ordered as: Route A (Central China, 21.59%) ≈ Route B (Mongolia Plateau/North China, 21.59%) > Route C (Korea/Japan, 20.45%) > Route E (South China Sea, 19.32%) > Route D (West Pacific, 17.05%), while their two-year averaged GEM concentrations at the Taiping Island were 1.54, 1.29, 1.26, 1.45, and 1.36 ng/m^3^, respectively (Table 2). Among the five transport routes, Routes A, B, and C most frequently occurred in winter and spring, while Routes D and E were mainly observed in summer and fall.

As illustrated in the fire maps of East and South Asia (see Fig. 6), the frequencies of open fires occurred in Central China, North China, South Korea and Japan were relatively higher in winter and spring than those in summer and fall. As the prevailing wind blew from the north toward the Taiping Island, the concentration of GEM increased accordingly. It was worth noting that the highest GEM concentration was observed for Route A in spring when Central China had the highest density of fire spots. Route E had the second highest GEM concentration that mostly occurred in summer. Backward trajectories showed that air masses were blown southwesterly from the southern SCS to the Taiping Island, which was most likely originated from the Kalimantan and Sumatra Islands where open fires burned vigorously (see Fig. 6), thus resulting in the second highest GEM concentration at the Taiping Island. The third highest GEM concentration was observed for Route D which passed through the Central Philippine Islands, where demonstrated relatively higher frequency of open fires in winter and followed by those in spring. Oppositely, the relatively low GEM concentrations at the Taiping Island were mostly observed for Routes B and C that occurred frequently in winter and spring. Although Routes B and C could apparently blow GEM from anthropogenic sources located at the Northeast Asia to the leeward northern South China Sea^22,26^, the concentrations of GEM measured at the Taiping Island were much lower than those at the Dongsha Island, and close to the GEM background level of Northern Hemisphere in all seasons. Therefore, we concluded that Asian Northern Monsoons derived from Mongolian high pressure systems seemed not strong enough to transport the polluted air masses down to the Taiping Island in the central SCS. On the contrary, the emissions of huge amounts of mercury from the neighboring regions such as the Taiwan Island, the Philippines Islands, the Kalimantan Island, and the Sumatra Islands (Malaysia and Indonesia) had dominant influences on the concentration of GEM at the Taiping Island.

### Correlation of atmospheric speciated mercury with biomass burning

Previous literature reported that levoglucosan is produced by the pyrolysis of plant cellulose through high-temperature combustion of plant debris and woods^41,42^. During the burning processes, levoglucosan was formed as an intermediate product which can be used as an indicator of biomass burning since cellulose was the main component of plant xylem. Moreover, K^+^ has also been characterized as another valuable indicator of biomass burning since K^+^ is highly abundant in the plant tissues^43^.

**Figure 8:**
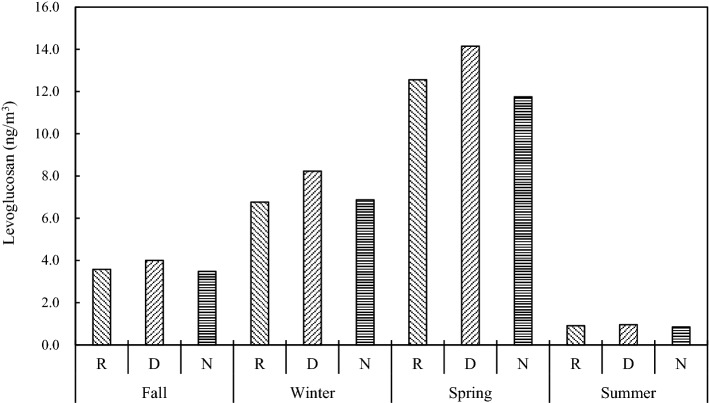
Seasonal variation of levoglucosan concentration in PM_2.5_ sampled at the Taiping Island in the South China Sea (R: regular sampling; D: daytime; N: nighttime).

The seasonal variation of levoglucosan concentration in PM_2.5_ sampled at the Taiping Island is shown in Table S1 and Fig. 8. Field measurement results indicated that high concentrations of levoglucosan in PM_2.5_ were commonly observed in spring and winter at the Taiping Island, implying that polluted air masses containing both levoglucosan emitted from biomass burning, which could be transported from Asian continent to the SCS. As illustrated in Fig. 6, the density of fire spots in China, India, the Indochina Peninsula, and the Philippines Islands surrounding the SCS increased significantly in winter and spring. On the contrary, the fire maps showed that the Taiping Island was mainly affected by biomass burning commonly occurred in East Malaysia and Indonesia (e.g. Sumatra and Kalimantan Islands), and the Indochina Peninsula in summer season (see Fig. 6) although the concentrations of levoglucosan in PM_2.5_ were relatively low at the Taiping Island (see Fig. 8). Both levoglucosan and gaseous mercury concentrations had similar trend at the Taiping Island, showing that gaseous mercury was also emitted from the upwind biomass burning sources.

**Figure 9:**
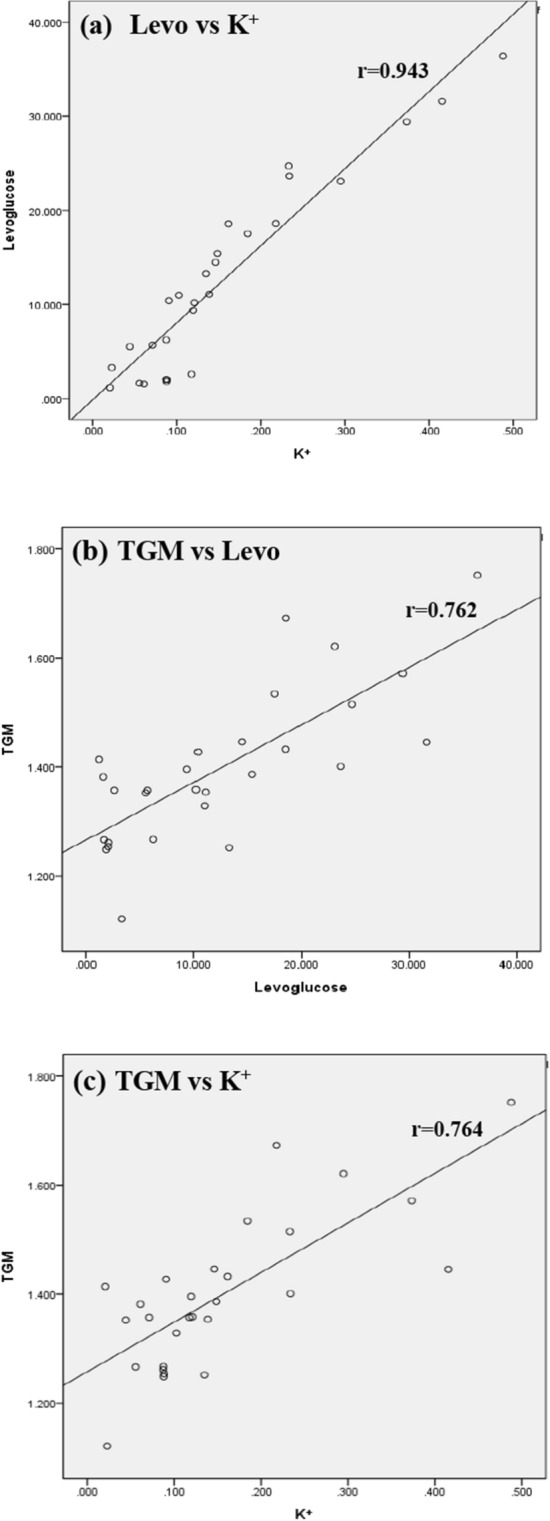
Scatter diagrams of levoglucosan versus K^+^ as well as TGM versus levoglucosan and K^+^ in PM_2.5_ at the Taiping Island in the South China Sea.

Furthermore, this study correlated atmospheric speciated mercury with PM_2.5_ and its chemical content of Levoglucosan and K^+^. The correlation coefficients and *p* values of atmospheric speciated mercury, PM_2.5_, Levoglucosan, and K^+^ are summarized in Table 3. The scatter diagrams of levoglucosan versus K^+^ in PM_2.5_ as well as TGM versus levoglucosan and K^+^ are illustrated in Fig. 9. It showed undoubtedly that levoglucosan had very strong correlation with K^+^ (r = 0.943, *p* < 0.01), indicating that both levoglucosan and K^+^ were emitted from much similar biomass burning sources. In terms of atmospheric speciated mercury, TGM strongly correlated with levoglucosan (r = 0.764, *p* < 0.01) and K^+^ (r = 0.758, *p* < 0.01), but weakly correlated with PM_2.5_ (r = 0.352, *p* < 0.01). The results confirmed that gaseous mercury (i.e. TGM) in the atmosphere of the Taiping Island was mainly emitted from the biomass burning sources, but was not from the industrial sources. Similarly, GOM and PHg also had moderate correlation with levoglucosan and K^+^ (r = 0.576–0.644, *p* < 0.01) (see Table 3).

**Table 3:**
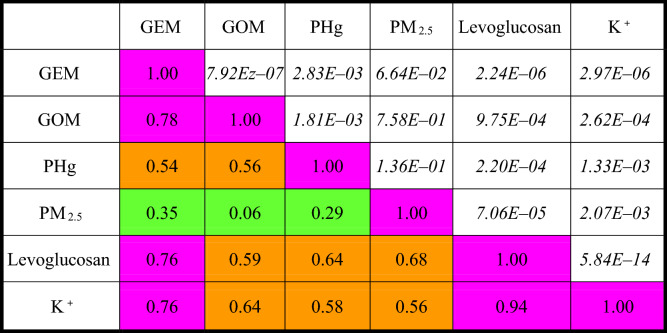
Correlation coefficients and *p* values of atmospheric speciated mercury, PM_2.5_, Levoglucosan, and K^+^.

*GEM* gaseous elemental mercury, *GOM* gaseous oxidized mercury, *PHg* particle-bound mercury, *RH* relative humidity, *WS* wind speed; Color Index of Correlation Coefficient (r): strong (purple, 0.70–1.00), moderate (brown, 0.40–0.69), and low (green, 0–0.39); Italic Numbers: *p* values.

Results obtained from this study were in accordance with previous reports^44–46^, indicating that gaseous mercury can be emitted from biomass burning of forests (^47,48^) and agricultural debris^46^. Clear evidences for long-range transport of atmospheric speciated mercury emitted from the biomass burning sources in the surrounding regions of the SCS towards the Taiping Island were obtained and confirmed in the present study, which also concurred with previous literature (Sigler et al. ^47^).

### Comparison with remote islands and coastal sites in East Asia

The concentrations of atmospheric speciated mercury measured in the present study were further compared with previous literature. Table 4 summarizes the concentrations of GEM, GOM, and PHg measured at the Taiping Island as well as other remote islands and coastal areas in the East Asia. It showed that the two-year averaged GEM concentrations at the Taiping Island were 1.37 ± 0.48 ng/m^3^, respectively (see Table 1), which were as high as the background GEM concentration (1.3–1.5 ng/m^3^) of the Northern Hemisphere. As far as other remote islands and coastal areas in the East Asia was concerned, the concentration of GEM at the Taiping Island was generally lower than those at other East Asian islands, but similar to that at the Nansha Islands^26^, indicating that mercury emissions from the surrounding countries of the SCS were much lower than other Asian remote islands and coastal areas.

**Table 4:** Comparison of atmospheric speciated mercury concentrations at remote islands and coastal sites in the East Asia.

Sampling sites	GEM (ng/m^3^)		GOM (pg/m^3^)	PHg (pg/m^3^)	References
**Taiwan (ROC)**					
Matsu Islands, Taiwan	Island	4.56 ± 0.30	–	170 ± 20	^49^
Penghu Islands, Taiwan	Island	4.60 ± 1.38	54 ± 37	210 ± 124	^50^
Penghu Islands, Taiwan	Island	3.28 ± 0.65	23.68 ± 10.22	260 ± 70	^25^
Penghu Islands, Taiwan	Island	2.82 ± 0.57	31.71 ± 1.85	250 ± 63	^51^
Dongsha Islands, Taiwan	Island	2.17 ± 0.09	–	–	^22^
Dongsha Islands, Taiwan	Island	2.10 ± 0.46	21.33 ± 8.11	110 ± 78	^51^
Nansha Islands, Taiwan	Island	1.34 ± 0.24	13.49 ± 3.17	100 ± 70	^51^
Taiping Island, Taiwan	Island	1.37 ± 0.48	13.42 ± 4.56	84 ± 27	This study
Taiwan Strait and South China Sea	Island	2.09 ± 0.42	35.27 ± 4.38	150 ± 70	^51^
Checheng, Taiwan	Coast	2.26 ± 0.68	19.57 ± 7.83	120 ± 35	^27^
Kaohsiung, Taiwan	Island	6.70 ± 1.40	142.63 ± 153.17	290 ± 210	^29^
Pingtung, Taiwan	Island	2.56 ± 0.92	24.05 ± 8.24	130 ± 40	^25^
**China**					
Yellow Sea and Bohai Sea, China (Spring)	Island	2.03 ± 0.72	2.5 ± 1.7	11.3 ± 18.5	^52^
Yellow Sea and Bohai Sea China (Fall)	Island	2.09 ± 1.58	4.3 ± 2.5	9.0 ± 9.0	^52^
South China Sea, China	Island	2.32 ± 2.62	–	–	^30^
Xiamen, China	Coast	3.50	174.41	61.05	^53^
**Japan**					
Tokaimura, Japan	Coast	3.78 ± 1.62	–	–	^54^
Okinawa Islands, Japan	Island	2.04 ± 0.38	4.5 ± 5.4	3.0 ± 2.5	^37^
Kyushu Islands, Japan	Island	2.33 ± 0.49	5.7 ± 9.4	10 ± 11	^55^
**Korea**					
Anmyun Island, Korea	Island	4.61 ± 2.21	–	–	^56^
Jeju Island, Korea	Island	3.85 ± 1.68	–	–	^57^
Chuncheon, Korea	Coast	2.12 ± 1.47	2.7 ± 2.7	3.7 ± 5.7	^58^
**The Philippines**					
Laoag, the Philippines	Coast	3.00 ± 0.69	26.69 ± 9.23	220 ± 46	^27^

Previous literature reported that remote islands and continental sites commonly have sparse population and limited emission sources, thus their mercury concentrations were close to the background level. Comparing with the remote continental sites such as Mt. Lulin^59^, Checheng^27^, and Dongsha Islands^26,59^ in Taiwan, and Mt. Changbai^60^ and Waliguan^30^ in China, we found that the concentration of GEM at the Taiping Island was relatively low. We further compared the measured atmospheric speciated mercury at the Taiping Island with Taiwan’s main islands including the Matsu Islands^49^, the Penghu Islands^25,26^, and the Dongsha Islands^22,26,50^. Very similarly, the concentrations of GEM at the Taiping Island were lower than those at those offshore islands since mercury emissions from Taiwan and China to these three offshore islands were much higher than those at the Taiping Island^50^. Although the concentrations of GEM at the Dongsha Islands were lower than those at the Penghu and Matsu Islands^49,50^, the Taiping Island had much lower GEM concentrations than the Dongsha Islands.

## Conclusions

The temporal variation, gas-particle partition, and potential origins of atmospheric speciated mercury at the Taiping Island in the central SCS were firstly investigated for both regular and intensive sampling. Two-year measurement data showed that the concentrations of atmospheric speciated mercury in cold seasons (winter and spring) were commonly higher than those in hot seasons (summer and fall). In terms of mercury species, total gaseous mercury, particularly GEM, dominated TAM and accounted for 86.2–98.5% of TAM at the Taiping Island. Particle-bounded mercury (PHg) accounted only for 1.5–13.8% of TAM with the highest percentage of 13.8% observed in winter.

The concentrations of atmospheric speciated mercury at the Taiping Island varied with season and were highly influenced by the transport of mercury emitted from biomass burning sources in the surrounding countries. Clustered transport routes showed that polluted air masses came mainly from the north routes (i.e. Routes A, B, and C) in winter and spring, while those in summer and fall came mostly from the east and the south routes (i.e. Routes D and E). The highest concentrations of GEM came from Route A and followed by Routes D and E, while the lowest GEM concentrations came from Routes B and C. This study revealed that the concentration of GEM highly correlated with those of levoglucosan and K^+^ in PM_2.5_, implying that atmospheric mercury was mainly emitted from biomass burning sources at the Philippines Islands in winter and spring as well as at the Kalimantan and Sumatra Islands in summer. It highly concurred with the seasonal variation of regional fire maps in the East and South Asia. Compared with other remote islands and coastal areas in the East Asia, the concentration of GEM observed at the Taiping Island was much lower and close to the background level of Northern Hemisphere.

## Supplementary Information

Supplementary Information
