# Supplementary material for: Temporal variation and potential origins of atmospheric speciated mercury at a remote island in South China Sea based on two-year field measurement data
Source: Sci Rep. 2021 Mar 11;11:5678. doi: 10.1038/s41598-021-84434-z (PMC7952567; doi:10.1038/s41598-021-84434-z)
Supplement: Supplementary file 1 — Supplementary Information [file 41598_2021_84434_MOESM1_ESM.docx]

**Temporal Variation and Potential Origins of Atmospheric Speciated Mercury at a Remote Island in South China Sea Based on Two-year Field Measurement Data**

**Supporting Information**

（two Table and two Figure included）

Ming-Jie Yeh^1^, Chung-Shin Yuan^1^*, Kuo-Ning Hung^1^, Iau-Ren Ie^1^,

Cheng-En Lee^1^, Kuan-Chen Chiang^1^, Ker-Yea Soong^2^

^1^Institute of Environmental Engineering, National Sun Yat-Sen University, Taiwan, ROC

^2^Institute of Marine Biology, National Sun-Yat Sen University, Taiwan, ROC

* To whom all correspondence should be addressed

Tel: +86-15260231263

Tel: +886-7-5252000 Ext. 4409; Fax: 886-7-52524409;

E-mail:ycsngi@nsysu.edu.tw

**Table S1**. Meteorological data measured at the Taiping Island during the sampling campaign in four seasons.

| **Years** | **Seasons** | **Air Temperature (℃)** | **Relative Humidity (%)** | **Precipitation (mm)** | **Wind Speed (m/s)** | **Wind Direction** |
| --- | --- | --- | --- | --- | --- | --- |
| **2017** | **Fall** | 28.2 | 82 | 1.8 | 5.9 | SW/NE |
| **2018** | **Winter** | 28.4 | 82 | 1.5 | 9.2 | NNE |
|  | **Spring** | 30.2 | 81 | 0.7 | 5.6 | ENE |
|  | **Summer** | 31.1 | 88 | 2.3 | 5.7 | SW |
|  | **Fall** | 28.3 | 86 | 79.4 | 4.2 | NNE |
| **2019** | **Winter** | 27.9 | 81 | - | 6.2 | ENE |
|  | **Spring** | 29.7 | 77 | 6.5 | 2.6 | ENE |
|  | **Summer** | 29.0 | 81 | 20.0 | 5.5 | SW |

- : no precipitation

**Table S2**. Concentrations of levoglucosan in PM_2.5_ at the Taiping Island during the sampling campaign in four seasons.

| **Species of Anhydrosugars** | **Sampling Modes** | **Fall** | **Winter** | **Spring** | **Summer** |
| --- | --- | --- | --- | --- | --- |
| **Levoglucosan (ng/m^3^)** | RS | 3.58 | 6.76 | 12.56 | 0.91 |
|  | IS-D | 4.00 | 8.23 | 14.15 | 0.96 |
|  | IS-N | 3.48 | 6.87 | 11.75 | 0.85 |
|  | Average | 3.69 | 7.29 | 12.82 | 0.91 |

RS: regular sampling for 24 hours (8:00-8:00); IS-D: intensive sampling in the daytime (8:00-20:00);

IS-N: intensive sampling at nighttime (20:00-8:00)


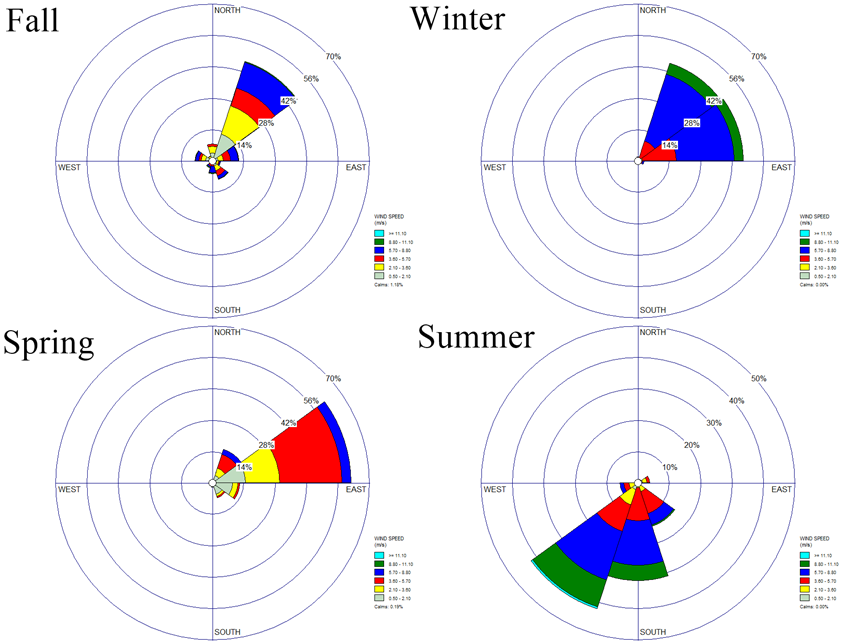


**Figure S1.** Wind roses at the Taiping Island during the sampling campaign in four seasons.

**Figure S2.** Calibration curves of (a) high- (ng) and (b) low-level (pg) mercury concentrations for GEM and GOM/PHg measurements, respectively..
